# Pathological components and CT imaging analysis of the area adjacent pleura within the pure ground-glass nodules with pleural deformation in invasive lung adenocarcinoma

**DOI:** 10.1186/s12885-022-10043-2

**Published:** 2022-09-06

**Authors:** Yining Jiang, Ziqi Xiong, Wenjing Zhao, Di Tian, Qiuping Zhang, Zhiyong Li

**Affiliations:** 1 Department of Radiology, the First Affiliated Hospital of Dalian Medical University, Dalian, China; 2 Department of Pathology, the First Affiliated Hospital of Dalian Medical University, Dalian, China

**Keywords:** Invasive lung adenocarcinoma, Pure ground-glass nodules, Pleural deformation, Pathology

## Abstract

**Background:**

Pleural deformation is associated with the invasiveness of lung adenocarcinoma(LAC). Our study focused on the pathological components of the area adjacent pleura in pulmonary pure ground-glass nodules(pGGNs) with pleural deformations(P-pGGNs) confirmed to be invasive LAC without visceral pleural invasion (VPI) pathologically.

**Methods:**

Computed tomography(CT) imaging features of nodules and pathological components of the area adjacent pleura were analyzed and recorded. Statistical analysis was performed for subgroups of P-pGGNs.

**Results:**

The 81 enrolled patients with 81 P-pGGNs were finally involved in the analysis. None of solid/micropapillary group and none of VPI was observed, 54 alveoli/lepidics and 27 acinar/papillarys were observed. In P-pGGN with acinar/papillary components of the area adjacent pleura, invasive adenocarcinoma (IAC) was more common compared to minimally invasive adenocarcinoma (MIA, 74.07% vs. 25.93%; *p* < 0.001). The distance in alveoli/lepidic group was significantly larger (1.50 mm vs. 0.00 mm; *p* < 0.001) and the depth was significantly smaller (2.00 mm vs. 6.00 mm; *p* < 0.001) than that in acinar/papillary group. The CT attenuation value, maximum diameter and maximum vertical diameter was valuable to distinguish acinar/papillary group form alveoli/lepidic group(*p* < 0.05). The type d pleural deformation was the common pleural deformation in IAC(*p* = 0.028).

**Conclusions:**

The pathological components of the area adjacent pleura in P-pGGN without VPI confirmed to be invasive LAC could included alveoli/lepidics and acinar/papillarys. Some CT indicators that can identify the pathological invasive components of the area adjacent pleura in P-pGGNs.

**Supplementary Information:**

The online version contains supplementary material available at 10.1186/s12885-022-10043-2.

## Background

Subpleural nodules or tumors contacting the visceral pleura, or appearing linear opacities that are vertical and intersect with the visceral pleura, may cause pleural deformation [1]. Pleural deformation can be directly detected on computed tomography (CT) by radiologists and has a variety of manifestations [2]. Previous reports have suggested that pleural deformations were associated with higher recurrence, all-cause mortality rate and poor prognosis [3, 4]. Pleural deformation can also be a powerful supportive tool for increasing the accuracy of early diagnosis of visceral pleural invasion(VPI) by non-small cell lung cancer (NSCLC) [5].

Previous studies suggested that pleural deformation was associated with the likelihood of invasiveness for lung adenocarcinoma (LAC) [6]. But in actual fact, not all pGGN with pleural deformation (P-pGGN) were IACs. There have been many instances of P-pGGN identified as MIAs [7]. In our previous research, 17.09% (47/275) MIAs and 19.27% (53/275) IACs with pleural deformation were observed in patients diagnosed as invasive LAC presenting as pGGN [8]. Evaluation of P-pGGNs in invasive adenocarcinoma would get further attention. Most published studies have shown that VPI could be observed in both MIA and IAC, but VPI rarely occurred in pGGN. Kim et al. [3] reported that VPI was observed in 15.25% (36/236) patients whose nodules were identified as detected as part-solid nodule with pleural deformation (P-PSN) and was observed in 0.00% (0/48) patients who detected as P-pGGN. Ahn et al. [9] reported that VPI was observed in 28.13% (36/128) patients who detected as P-PSN and was observed in 0.00% (0/24) patients who detected as P-pGGN. Zhao et al. [2] investigated the risk of VPI in P-pGGNs and indicated that this indolent tumour generally did not invade the pleura. Pleural deformation was a powerful CT feature suggesting the potential VPI, but when invasive LAC manifested P-pGGN, the VPI was rarely observed. Detailed evaluation of pleural deformation requires to be further explored pathologically.

According to the IASLC pathology committee: lepidic growth was proposed as well differentiated; acinar or papillary growth was proposed as moderately differentiated; more high-grade patterns was proposed as poor differentiated [10]. Lepidic growth is a noninvasive component of LAC. At present, it is still unclear whether various pleural deformations on CT imaging are bound up with variously pathological components of the area adjacent pleura. Therefore, the significance of the pathological components of the area adjacent pleura within P-pGGNs deserves further study. Further understanding the relationship between pleural deformation on HRCT and the pathological components of the area adjacent pleura could promote the individualized follow-up strategy of P-pGGNs. Herein, in this retrospective study, our intention is to analysis the pathological components of the area adjacent pleura and the CT manifestation of pleural deformations in P-pGGNs confirmed to be invasive lung adenocarcinoma without VPI pathologically, as well as to explore the relationship between them.

## Methods

### Patients

The patients with GGNs (from January 2017 to December 2018) were pulled from our institution’s picture archiving and communication system (PACS, DJ Health Union Systems Corporation) under the approval of the institutional review board with a waiver allowing the data to be used retrospectively without informed consent. Inclusion criteria: (A) preoperative chest HRCT examinations performed within 2 weeks; (B) MIA or IAC confirmed pathologically after thoracic resection which tissue specimens were complete and reviewable; (C) P-pGGNs located next to the pleura on the preoperative HRCT, defined as pGGNs (window width, 1,500 HU; window level, -600 HU) contacted or connected to the pleural surface including the non-interlobar and interlobar pleura. Exclusion criteria: (A) underwent tumour therapy (radiotherapy, chemotherapy, etc.), puncture biopsy, or surgical resection were performed before the HRCT examinations; (B) impaired image sequences in PACS; (C) detectable soft-tissue attenuation inside the tumour on the mediastinal window images (window width, 400 HU; window level, 40 HU). (D) VPI cases observed during pathologic review. The demographic (age and sex) and smoking history characteristics of all P-pGGNs were recorded.

### CT imaging parameters

Unenhanced chest CT examinations for all enrolled patients were obtained before P-pGGNs resection via scanning machines (Optima CT660, Discovery CT750 HD, Revolution CT or LightSpeed16 from General Electric, SOMATOM Perspective or Emotion 16 from Siemens, Brilliance 16P from Philips). All patients underwent chest examinations with their hands in a supine position on either side of the head from the lung apex to the lung base. All patients were instructed to hold their breath for the whole scan period in a deep-inhalation state. Though various chest CT imaging protocols were used in this study, all examinations were performed with contiguous 1.00–1.50 mm axial sections and 1.00–1.50 mm slice intervals and 0.625–1.50 mm section thickness after reconstruction. Imaging parameters as follows: a matrix of 512 × 512, a tube current of 170–200 mA, a tube voltage of 120 kVp, rotation times of 0.5–0.6 s, and a full field of view. Data were reconstructed with a lung kernel algorithm.

### Review of pathological sections

All histological specimens were reviewed by a senior pathologist (Z.Q.P. with 30 years of experience) who blinded to the primary pathological diagnosis. The comparison between the reviewed and primary pathological diagnoses were recorded. Pathological diagnoses were classified in accordance with the lung tumor classification suggested by the IASLC/ATS/ERS in 2011 [11].

**Fig. 1:**
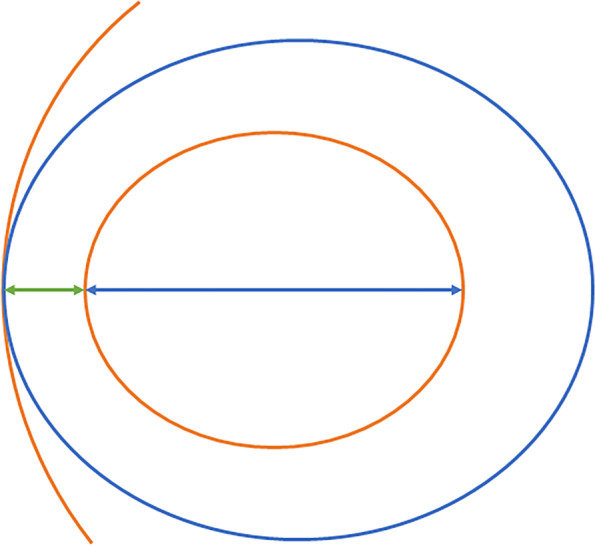
The detailed schematic diagram of measurement. The orange arc represents the pleura, the blue regular oval represents a P-pGGN, the orange regular oval represents the largest invasive component area which closest to the pleura, the green two-way arrow represents the closest distance between the largest invasive component area and the pleura, and the blue two-way arrow represents the depth of the largest invasive component area

All histological specimens were formalin fixed and hematoxylin–eosin stained. The observation of pleura in all sections were based on hematoxylin–eosin staining. Ki-67 staining was performed with Ki-67 protein antibody (Santa Cruz Biotechnology, Santa Cruz, CA, USA) at a dilution of 1:100. Ki-67, a protein expressed in proliferating cells, has been used to identify high risk people group in LAC. Cells with brown-stained nuclei are considered positive cells, which are associated with cell proliferation. Increased Ki-67 expression is also significantly associated with lymph node metastasis and a poorer prognosis of LAC [12, 13]. The microscopic features of pathological components of the area adjacent pleura within lesion were categorized by using Olympus microscope as: alveoli/lepidic, acinar/papillary and solid/micropapillary type [10]. Distance was defined as the shortest distance between the largest invasive component area and the pleura; Depth was defined as the depth of the largest invasive component area (the vertical distance between the point closest to the pleura and the point farthest from the pleura within invasive component area). Distance and depth were calculated microscopically. The detailed schematic diagram of measurement is shown in Fig. 1. The largest area closest to the pleura was selected when the invasive components were scattered. The subpleural Ki-67 staining were counted. The range of Ki-67 counting was from visible pleural radiation to the center of the lesion and the average was recorded.

### Image characteristics analysis

All HRCT images of P-pGGNs were observed by two thoracic radiologists (a junior radiologist and a senior thoracic radiologist with 20 years of experience) who blinded to the pathological results in the lung window (window width, 1500 HU; window level, -600 HU). The following imaging features were recorded and the discrepancies in the observation between two radiologists were resolved by consensus: (1) tumor location; (2) shape: round and oval, irregular; (3) tumor-lung interface: clear or not; (4) lobulation: defined as a portion edge of the nodule was wavy or fan-shaped; (5) vacuoles: defined as 1.00–3.00 mm cystic transparency of air attenuation within nodules; (6) airbronchogram: defined as the lucency along the regular bronchial wall within the nodules. The maximum axial layer selected from the HRCT image on the lung window: (7) the maximum diameter (MD); (8) the maximum vertical diameter (MVD) of the MD; (9) CTv: the CT attenuation value. When measuring all quantitative CT features, it should be noted that region of interest of P-pGGNs were delineated by a unregular curve and large vessels, bronchus and vacuoles should be avoided, when they existed in the measurement layer.

**Fig. 2:**
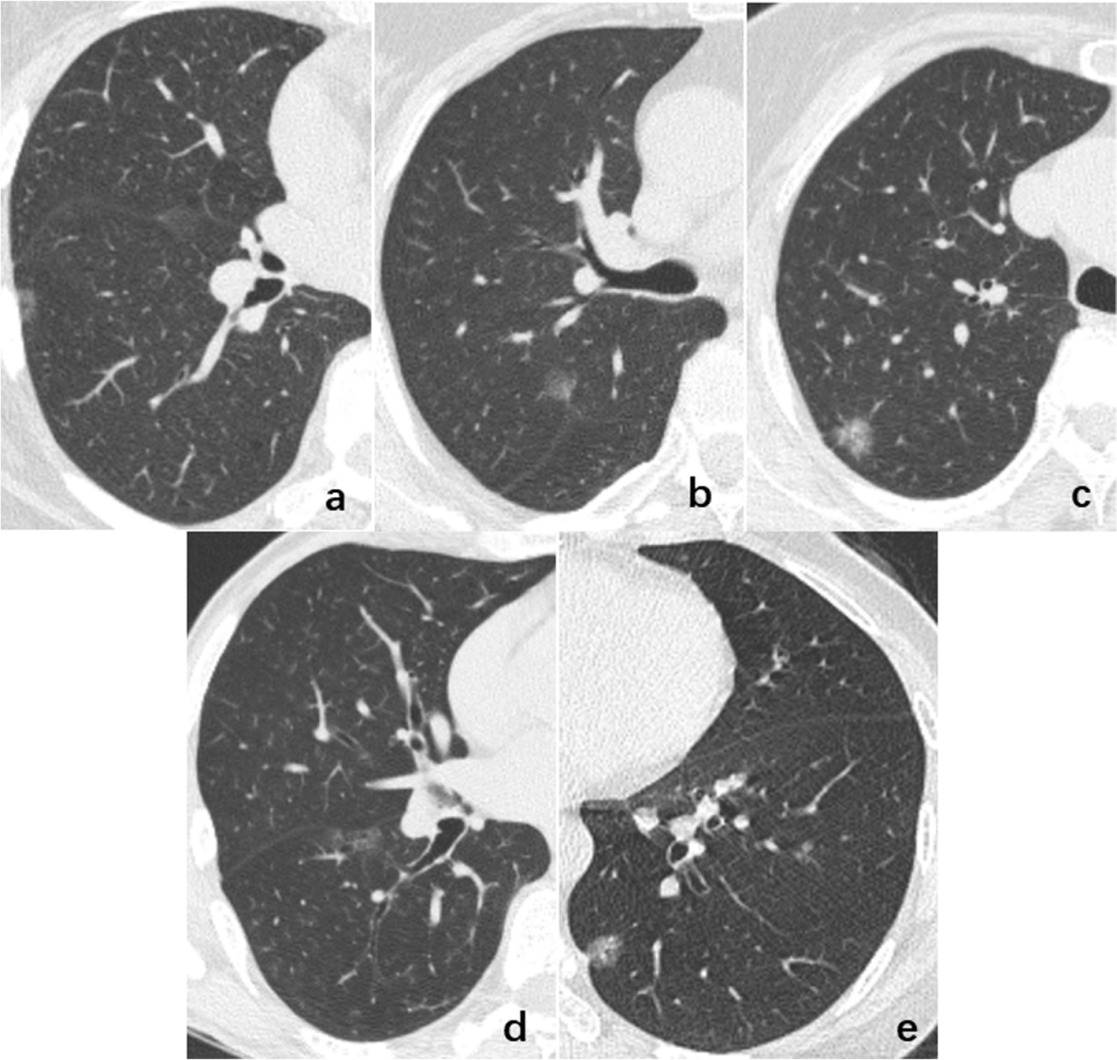
The representative images of the relationship between P-pGGNs and pleura. The type **a**, pleural attachment without pleural distortion; type **b**, pleural tag with pleural folding; type **c**, pleural tag without pleural folding; type **d**, pleural retraction showing enfoldment of the pleura into the tumour; type **e**, tentiform indrawing of the pleura toward the tumour

The five subgroups were allocated according to pleural deformation [2]: Type a, pleural attachment without pleural distortion; Type b, pleural tag with pleural folding; Type c, pleural tag without pleural folding; Type d, pleural retraction showing enfoldment of the pleura into the tumour; Type e, tentiform indrawing of the pleura toward the tumour (Fig. 2).

### Statistical analysis

The normal distribution was confirmed by the Kolmogorov–Smirnov test. The T-tests were applied for normally distributed features expressing as the mean ± standard deviation, Mann–Whitney U tests were applied for nonnormally distributed features expressing as the median and quartile. The Kappa analysis was used to assess agreement for P-pGGNs primary and reviewed pathological diagnoses. Pearson’s chi-square tests or Fisher’s exact tests were applied for categorical variables. All statistical analyses for the present study were executed by SPSS (version 26.0, IBM, Armonk, NY, USA). A two-tailed *P*-value < 0.05 was considered to indicate statistical significance.

## Results

**Fig. 3:**
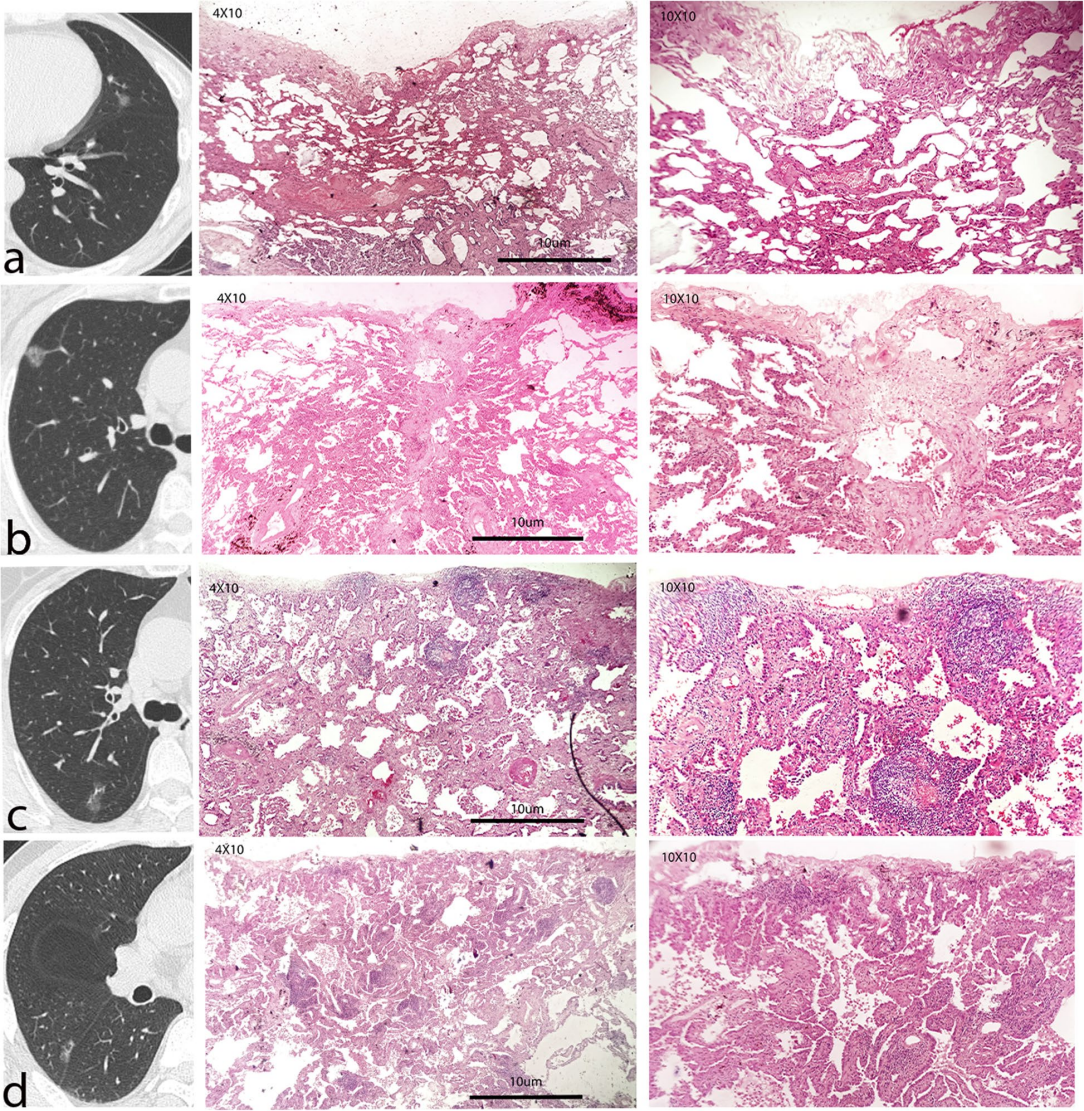
The P-pGGNs representative CT images and microscopic subpleural pathological basis images. The line a presented an axial CT image and hematoxylin–eosin stained sections image of P-pGGN with a pleural tag without pleural folding in a 56-year-old male, showing that the tissue adjacent to the pleura was alveoli. The line b presented an axial CT image and hematoxylin–eosin stained sections image of P-pGGN with a pleural attachment without pleural distortion in a 68-year-old female, showing that the tissue adjacent to the pleura was lepidic. The line c presented an axial CT image and hematoxylin–eosin stained sections image of P-pGGN with a pleural retraction showing enfoldment of the pleura into the tumour in a 67-year-old female, showing that the tissue adjacent to the pleura was acinar. The line d presented an axial CT image and hematoxylin–eosin stained sections image of P-pGGN with a pleural retraction showing enfoldment of the pleura into the tumour in a 50-year-old male, showing that the tissue adjacent to the pleura was papillary

The inclusion and exclusion criteria were applied to 276 patients with GGN, and a total of 103 P-pGGNs were included for pathological review. During the pathological reexamination, 22 cases identified as unable to complete microscopic measurement were excluded, including 2 bronchiolar adenomas [14], 7 AISs and 13 unclear pleural. Therefore, 81 enrolled patients (median age 60.00, range 49.50–64.00) with 81 P-pGGNs were finally involved in the analysis. None of solid/micropapillary group and none of VPI was observed, 54 alveoli/lepidics and 27 acinar/papillarys were observed (Fig. 3). Details of the pathological review were obtained in the Additional file 1 Table S1.

In P-pGGN with acinar/papillary components of the area adjacent pleura, IAC was more common compared to MIA (74.07% vs. 25.93%; *p* < 0.001). The distance in alveoli/lepidic group was significantly larger (1.50 mm vs. 0.00 mm; *p* < 0.001) and the depth was significantly smaller (2.00 mm vs. 6.00 mm; *p* < 0.001) than that in acinar/papillary group. There was no difference in CT morphological manifestations between alveoli/lepidic group and acinar/papillary group for all cases (*p* > 0.05, Table1). In CT quantitative features, CTv, MD and MVD were significantly different between in alveoli/lepidic group and acinar/papillary group (*p* < 0.05). Details are shown in Table 1.

**Table 1:** Differential analysis between alveoli/lepidic and acinar/papillary according to the pathological components of the area adjacent pleura and CT manifestation

Variable	Alveoli/Lepidic(*n* = 54)	Acinar/Papillary(*n* = 27)	Total(*n* = 81)	*P* value
**Age** Median (25th to 75th percentile) years	58.50(47.50, 64.25)	61.00(51.00, 63.00)	60.00(49.50, 64.00)	0.790
**Sex**/Male, No. (%)	17(31.48%)	7(25.93%)	24(29.63%)	0.797
**Smoking history**, No. (%)	4(7.41%)	2(7.41%)	6(7.41%)	1.000
**Pathological types**, No. (%)				< 0.001
IAC	11(20.37%)	20(74.07%)	31(38.27%)	
MIA	43(79.63%)	7(25.93%)	50(61.73%)	
**Ki-67**, No. (%)				0.244
< 10%	48(94.12%)	20(83.33%)	68(90.67%)	
≥ 10%	3(5.88%)	4(16.67%)	7(9.33%)	
Absence	3	3	6	
**Distance**(mm), median (25th – 75th percentile)	1.50(1.00, 2.00)	0.00(0.00, 0.00)	1.00(0.00, 2.00)	< 0.001
**Depth**(mm) median (25th – 75th percentile)	2.00(0.50, 3.25)	6.00(4.00, 10.00)	3.00(1.00, 6.00)	< 0.001
**Tumor location**, No. (%)				0.247
Right upper lobe	16(29.63%)	15(55.55%)	31(38.27%)	
Right middle lobe	5(9.26%)	2(7.41%)	7(8.64%)	
Right lower lobe	12(22.22%)	5(18.52%)	17(20.99%)	
Left upper lobe	14(25.93%)	3(11.11%)	17(20.99%)	
Left lower lobe	7(12.96%)	2(7.41%)	9(11.11%)	
**Shape**, No. (%)				1.000
Irregular	20(37.04%)	10(37.04%)	30(37.04%)	
Round and oval	34(62.96%)	17(62.96%)	51(62.96%)	
**Lobulation**, No. (%)				0.098
Absent	19(35.19%)	15(55.56%)	34(41.98%)	
Presence	35(64.81%)	12(44.44%)	47(58.02%)	
**Vacuole**, No. (%)				1.000
Absent	40(74.07%)	20(74.07%)	60(74.07%)	
Presence	14(25.93%)	7(25.93%)	21(25.93%)	
**AirBronchogram**, No. (%)				0.321
Absent	45(83.33%)	25(92.59%)	70(86.42%)	
Presence	9(16.67%)	2(7.41%)	11(13.58%)	
**Pleural deformation**, No. (%)				0.157
Type a	11(20.37%)	7(25.93%)	18(22.22%)	
Type b	7(12.96%)	3(11.11%)	10(12.35%)	
Type c	13(24.07%)	3(11.11%)	16(19.75%)	
Type d	17(31.48%)	14(51.85%)	31(38.27%)	
Type e	6(11.11%)	0(0.00%)	6(7.40%)	
**CTv** (HU)	-634.15(-676.02, -590.10)	-582.94(-652.12, -524.14)	-618.26(-672.59, -577.62)	0.024
**MD** (mm)	13.75(11.18, 17.93)	18.20(14.30, 23.20)	14.80(11.50, 19.40)	0.001
**MVD** (mm)	10.30(8.06, 13.38)	15.00(11.00, 17.30)	11.40(8.66, 15.65)	0.002

Distance: The distance between invasive components area and non-interlobar pleura; Depth: Depth is defined as the vertical distance between the point closest to the non-interlobar pleura and the point farthest from the non-interlobar pleura; CTv refers to the CT attenuation value on the maximum axial layer; MD refers to the maximum diameter on the maximum axial layer; MVD refers to the maximum vertical diameter of the maximum diameter on the maximum axial layer

In both IAC and MIA subgroups, distance still showed statistical differences between the alveoli/lepidic and acinar/papillary groups, but only in MIA subgroup, depth was smaller in alveoli/lepidic than that in acinar/papillary (1.50 mm vs. 2.50 mm; *p* < 0.05). For the IAC subgroup, the most common pleural deformation in acinar/papillary goup was the type d, in alveoli/lepidic group was the type c (*p* < 0.05). MD were significantly different between in alveoli/lepidic group and acinar/papillary group in the MIA subgroup, (*p* < 0.05). Details are shown in Table 2.

**Table 2:** Differential analysis in IAC and MIA according to the pathological components of the area adjacent pleura and CT manifestation

Variable	IAC				MIA			
	Alveoli/Lepidic (*n* = 11)	Acinar/Papillary (*n* = 20)	Total (*n* = 31)	*P* ^δ^value	Alveoli/Lepidic (*n* = 43)	Acinar/Papillary (*n* = 7)	Total (*n* = 50)	*P*^ε^value
**Age** Median (25th to 75th percentile) years	55.00(48.00, 63.00)	61.00(51.75, 64.00	60.00(49.00,64.00)	0.353	60.00(46.00, 66.00)	54.00(51.00,63.00)	59.50(50.50,64.25)	0.541
**Sex/Male**, No. (%)	3(27.27%)	4(20.00%)	7(22.58%)	0.676	14(32.56%)	3(42.86%)	17(34.00%)	0.677
**Smoking history**, No. (%)	2(18.18%)	2(10.00%)	4(12.90%)	0.601	2(4.65%)	0(0.00%)	2(4.00%)	0.737
**Subpleural histologic patterns**, No. (%)				–				–
Alveoli/lepidic	11(100.00%)	–	11(35.48%)		43(100.00%)	–	43(86.00%)	
acinar/papillary	–	20(100.00%)	20(64.52%)		–	7(100.00%)	7(14.00%)	
**Ki-67**, No. (%)				1.000				1.000
< 10%	9(81.82%)	13(76.47%)	22(78.57%)		39(97.50%)	7(100.00%)	46(97.87%)	
≥ 10%	2(18.18%)	4(23.53%)	6(21.43%)		1(2.50%)	0(0.00%)	1(2.13%)	
Absence	0	3	3		3	0	3	
**Distance**(mm), median (25th – 75th percentile)	1.00(0.50, 3.00)	0.00(0.00, 0.00)	0.00(0.00, 1.00)	< 0.001	1.50(1.00, 2.00)	0.00(0.00, 0.00)	1.00(0.50, 2.00)	< 0.001
**Depth**(mm) median (25th – 75th percentile)	6.50(6.00, 9.00)	7.00(5.00, 12.75)	7.00(5.00, 10.00)	0.573	1.50(0.50, 2.00)	2.50(2.00, 7.00)	1.50(0.50, 2.63)	0.044
**Tumor location**, No. (%)				0.136				0.672
Right upper lobe	2(18.18%)	11(55.00%)	13(41.94%)		14(32.56%)	4(57.14%)	18(36.00%)	
Right middle lobe	0(0.00%)	1(5.00%)	1(3.23%)		5(11.63%)	1(14.29%)	6(12.00%)	
Right lower lobe	5(45.45%)	4(20.00%)	9(29.03%)		7(16.28%)	1(14.29%)	8(16.00%)	
Left upper lobe	4(36.36%)	3(15.00%)	7(22.58%)		10(23.26%)	0(0.00%)	10(20.00%)	
Left lower lobe	0(0.00%)	1(5.00%)	1(3.23%)		7(16.28%)	1(14.29%)	8(16.00%)	
**Shape**, No. (%)				1.000				1.000
Irregular	4(36.36%)	8(40.00%)	12(38.71%)		16(37.21%)	2(28.57%)	18(36.00%)	
Round and oval	7(63.64%)	12(60.00%)	19(61.29%)		27(62.79%)	5(71.43%)	32(64.00%)	
**Lobulation**, No. (%)				0.716				0.234
Absent	5(45.45%)	11(55.00%)	16(51.61%)		14(32.56%)	4(57.14%)	18(36.00%)	
Presence	6(54.55%)	9(45.00%)	15(48.39%)		29(67.44%)	3(42.86%)	32(64.00%)	
**vacoule**, No. (%)				1.000				1.000
Absent	9(81.82%)	15(75.00%)	24(77.42%)		31(72.09%)	5(71.43%)	36(72.00%)	
Presence	2(18.18%)	5(25.00%)	7(22.58%)		12(27.91%)	2(28.57%)	14(28.00%)	
**AirBronchogram**, No. (%)				0.115				1.000
Absent	8(72.73%)	19(95.00%)	27(87.10%)		37(86.05%)	6(85.71%)	43(86.00%)	
Presence	3(27.27%)	1(5.00%)	4(12.90%)		6(13.95%)	1(14.29%)	7(14.00%)	
**Pleural deformation**, No. (%)				0.033				0.359
Type a	1(9.09%)	3(15.00%)	4(12.90%)		10(23.26%)	4(57.14%)	14(28.00%)	
Type b	4(36.36%)	3(15.00%)	7(22.58%)		3(6.98%)	0(0.00%)	3(6.00%)	
Type c	5(45.45%)	3(15.00%)	8(25.81%)		8(18.60%)	0(0.00%)	8(16.00%)	
Type d	1(9.09%)	11(55.00%)	12(38.71%)		16(37.21%)	3(42.86%)	19(38.00%)	
Type e	0(0.00%)	0(0.00%)	0(0.00%)		6(13.95%)	0(0.00%)	6(12.00%)	
**CTv (HU)**	-577.67(-608.57, -540.99)	-572.89(-634.41, -515.82	-575.57(-632.47, -518.47)	0.919	-654.24(-685.21, -606.12)	-632.84(-731.91, -599.28)	-647.94(-690.37, -605.85)	0.978
**MD (mm)**	14.60(13.10, 20.90)	17.50(13.78, 24.88)	16.80(13.20,23.90)	0.495	13.30(10.40, 17.90)	18.40(17.70,21.20)	14.10(10.75,18.43)	0.007
**MVD (mm)**	11.20(8.46,15.40)	14.95(11.25, 17.60)	12.80(10.60,17.30)	0.193	10.10(7.96, 13.10)	15.00(10.90,17.30)	10.45(7.98, 14.18)	0.065

P^δ^: The P^δ^ value was calculated by comparing the Alveoli/Lepidic group and Acinar/Papillary group in IAC; P^ε^: The P^ε^ value was calculated by comparing the Alveoli/Lepidic group and Acinar/Papillary group in MIA; Distance: The shortest distance between the largest invasive component area and the non-interlobar pleura; Depth: Depth is defined as the depth of the largest invasive component area (the vertical distance between the point closest to the non-interlobar pleura and the point farthest from the non-interlobar pleura); CTv refers to the CT attenuation value on the maximum axial layer; MD refers to the maximum diameter on the maximum axial layer; MVD refers to the maximum vertical diameter of the maximum diameter on the maximum axial layer

Compared with MIA subgroup, distance was smaller in IAC subgroup(0.00 mm vs.1.00 mm; *p* < 0.001); however, depth was larger in IAC (7.00 mm vs. 1.50 mm; *p* < 0.001). The pleural deformation, CTv, MD and MVD were significantly different between MIA and IAC subgroup (*p* < 0.05). The higher proliferation index Ki-67 proportion (Ki-67 ≥ 10%) showed a higher probability in IAC (21.43% vs. 2.13%; *p* = 0.010). Details were shown in Additional file 1 Table S2.

In both of non-interlobar and interlobar pleural subgroups, acinar/papillary components of the area adjacent pleura were significantly more common in IAC than MIA (*p* < 0.05). Both distance and depth were significantly different between alveoli/lepidic group and acinar/papillary group (*p* < 0.05). The MD and MVD in acinar/papillary group were significantly higher than that in alveoli/lepidic group for both subgroups. In addition, for P-pGGNs related to non-interlobar pleura, the pleural deformation was significantly different between alveoli/lepidic group and acinar/papillary group (*p* < 0.05). The CTv was significantly higher in acinar/papillary group than that in alveoli/lepidic group for P-pGGNs related to interlobar pleura (-599.28HU vs. -635.01HU, *p* = 0.040). Analysis details of P-pGGNs in non-interlobar and interlobar pleura could be seen in Additional file 1 Table S3.

## Discussion

In this article, we analyzed the pathological components of the area adjacent pleura and CT manifestation of pleural deformations in P-pGGNs diagnosed as invasive lung adenocarcinoma without VPI pathologically and tried to identify P-pGGNs of different pathological component types of the area adjacent pleura on CT images for the first time. We found that invasive LAC may appear pleural deformation similarly on CT, the pathological components of the area adjacent pleura could be different. Some CT indicators may help to identify the pathological invasive components of the area adjacent pleura in P-pGGNs.

The best time to eliminate tumor is to remove it before metastatic dissemination occurs, but early cancer diagnosis is limited by the sensitivity of clinical examination [15]. Improvements in noninvasive biomarkers of CT imaging that can distinguish early tumour from more invasive cases such as P-pGGN of VPI may occur in the future, will constantly bring down the detection limit. Kudo et al. [16] hypothesized based on physical movement of malignant cells that the visceral pleural is very rich in lymphatic vessels, with an intercommunicating “network” arranged over the lung surface. Lung tumour in a subpleural location can invades the pleura in a rapid speed and disseminate tumour cells throughout the pleural cavity, with subsequent systematic dissemination through subpleural lymphatics connecting with the pleural space. Therefore, we believe that P-pGGN of invasive components of the area adjacent pleura may be more likely to invade the pleura than P-pGGN of stable component of the area adjacent pleura. The non-invasive component of the area adjacent pleura probably plays a protective role to some extent. Study also had implicated among predominantly invasive LAC, the malignant potential was higher for pure invasive tumors without lepidic growth, and the prognosis was poorer than for partially invasive tumors with lepidic growth [17]. Although none of solid/micropapillary was observed in our study, we found that P-pGGN of acinar/papillary components of the area adjacent to the pleura were not uncommon, and IAC was the main histologic type. Therefore, in the process of clinical imaging diagnosis, we should not only pay attention to the identification of IAC from MIA, but also pay attention to the identification of acinar/papillary from alveoli/lepidic components of the area adjacent pleura.

Most of the previous studies analyzed the risk of patients with lung adenocarcinoma based on the primary histological component or the proportion of second most predominant component [18, 19]. Our study was based on the basic distribution of pathological components of the area adjacent pleura, including pathological components type of the area adjacent pleura within lesion, the shortest distance between the largest invasive component area and the pleura, the depth of the largest invasive component area. We found that when the pathological components of the area adjacent pleura were acinar/papillary, the size of the invasive components was not small, which to some extent indicates that this type of P-pGGN is risky and worthy of attention.

Previous articles mentioned pGGNs with irregular shapes, lobulation, larger size, higher attenuation tended to IAC [20, 21], in our study, it was found that semantic features played little role in identifying acinar/papillary components of the area adjacent pleura. However, CT quantitative features are still statistically valuable features. Bian et al. [22] also found that in tumors ≤ 3 cm, lepidic component at the tumor margin were significantly associated with histologic lepidic subtype (*P* < 0.05), which was similarly with our results. The larger size and higher attenuation value were still valuable indicators suggesting that pathological components of the area adjacent pleural might be acinar/papillary.

There still existed some limitations in our study. Because this was a single center retrospective study, the rigorous inclusion process made the sample size unsatisfactory. The association between patient outcomes and pathological findings was not searched and statistically analyzed in this study, which will be further analyzed in future studies. Besides, elastic staining was absent during our pathological review. Clear pleura and well stained sections were selected, which might miss the subpleural pathological information of other parts within the nodule. The VPI lesions were not enrolled for the pathological components of the area adjacent pleural and image control study. High-throughput quantitative analysis of P-pGGN three-dimensional volume may improve the comparative study of imaging and pathology. Further prospective large-sample study and logistic multivariate regression analysis are candidates to find the potential CT indicators to pathological components of the area adjacent pleural.

## Conclusion

The pathological components of the area adjacent pleura in P-pGGN without VPI confirmed to be invasive LAC could included alveoli/lepidics and acinar/papillarys. Some CT indicators that can identify the pathological invasive components of the area adjacent pleura in P-pGGNs.

## Supplementary Information

**Additional file 1:** **TableS1.** Detailed comparisons between the reviewed and primary pathologicaldiagnosis. **TableS2.** Differential analysisbetween IAC and MIA according to the pathologicalcomponents of the area adjacent pleura and CT manifestation. **TableS3.** Differential analysis between non-interlobar pleura and interlobar pleura according to the pathological components of the area adjacent pleura and CT manifestation.

## Data Availability

All data generated or analysed are included in this article [and its supplementary information files].

## Abbreviations

CT: Computed tomography
pGGN: Pulmonary pure ground-glass nodule
HRCT: High-resolution computed tomography
AIS: Adenocarcinoma in situ
MIA: Minimally invasive adenocarcinoma
IAC: Invasive adenocarcinoma
LAC: Lung adenocarcinoma
P-pGGN: PGGN with pleural deformation
VPI: Visceral pleural invasion
P-PSN: Part-solid nodule with pleural deformation
PACS: Picture archiving and communication system
HE: Hematoxylin–eosin
MD: The maximum diameter
MVD: The maximum vertical diameter
CTv: The CT attenuation value
