# Supplementary material for: Pathological components and CT imaging analysis of the area adjacent pleura within the pure ground-glass nodules with pleural deformation in invasive lung adenocarcinoma
Source: BMC Cancer. 2022 Sep 6;22:958. doi: 10.1186/s12885-022-10043-2 (PMC9447332; doi:10.1186/s12885-022-10043-2)
Supplement: Supplementary file 1 — Additional file 1: TableS1. Detailed comparisons between the reviewed and primary pathologicaldiagnosis. TableS2. Differential analysisbetween IAC and MIA according to the pathologicalcomponents of the area adjacent pleura and CT manifestation. TableS3. Differential analysis between non-interlobar pleura and interlobar pleura according to the pathological components of the area adjacent pleura and CT manifestation. [file 12885_2022_10043_MOESM1_ESM.docx]

The comparisons between the reviewed and primary pathological diagnosis were presented in Appendices table A1. Kappa analysis was carried to assess agreement for P-pGGNs diagnosis. The agreement between the reviewed and primary pathological diagnoses was ordinary, with a κ value of 0.280 (95% confidence interval [CI]: 0.155–0.405, p < 0.001). Agreement was seen in 57 P-pGGNs, and disagreement in 46 P-pGGNs.

Table S1. Detailed comparisons between the reviewed and primary pathological diagnosis

|  | | **Reviewed pathological diagnosis** | | | | | Total |
| --- | --- | --- | --- | --- | --- | --- | --- |
|  |  | Unclear pleura | bronchiolar adenoma | AIS | MIA | IAC |  |
| **Primary pathological diagnosis** | MIA | 2 | 2 | 6 | 30 | 4 | 44 |
|  | IAC | 11 | 0 | 1 | 20 | 27 | 59 |
| Total | | 13 | 2 | 7 | 50 | 31 | 103 |
| AIS, adenocarcinoma in situ; MIA, minimally invasive adenocarcinoma; IAC, invasive adenocarcinoma. | | | | | | | |

Table S2. Differential analysis between IAC and MIA according to the pathological components of the area adjacent pleura and CT manifestation.

| variables | IAC(n = 31) | MIA(n = 50) | Total | P value |
| --- | --- | --- | --- | --- |
| **Age**  Median (25th to 75th percentile) years | 60.00(49.00, 64.00) | 59.50(50.50, 64.25) | 60.00(49.50, 64.00) | 0.998 |
| **Sex/Male**, No. (%) | 7(22.58%) | 17(34.00%) | 24(29.63%) | 0.324 |
| **Smoking history**, No. (%) | 4(12.90%) | 2(4.00%) | 6(7.41%) |  |
| **Subpleural** **histologic patterns**, No. (%) |  |  |  | < 0.001 |
| **Alveoli/Lepidic** | 11(35.48%) | 43(86.00%) | 54(66.67%) |  |
| **Acinar/Papillary** | 20(64.52%) | 7(14.00%) | 27(33.33%) |  |
| **Ki-67**, No. (%) |  |  |  | 0.010 |
| < 10% | 22(78.57%) | 46(97.87%) | 68(90.67%) |  |
| ≥10% | 6(21.43%) | 1(2.13%) | 7(9.33%) |  |
| Absence | 3 | 3 | 6 |  |
| **Distance**(mm), median (25th - 75th  percentile) | 0.00(0.00, 1.00) | 1.00(0.50, 2.00) | 1.00(0.00, 2.00) | < 0.001 |
| **Depth**(mm) median (25th - 75th percentile) | 7.00(5.00, 10.00) | 1.50(0.50, 2.63) | 3.00(1.00, 6.00) | < 0.001 |
| **Tumor location**, No. (%) |  |  |  | 0.193 |
| Right upper lobe | 13(41.94%) | 18(36.00%) | 31(38.27%) |  |
| Right middle lobe | 1(3.23%) | 6(12.00%) | 7(8.64%) |  |
| Right lower lobe | 9(29.03%) | 8(16.00%) | 17(20.99%) |  |
| Left upper lobe | 7(22.58%) | 10(20.00%) | 17(20.99%) |  |
| Left lower lobe | 1(3.23%) | 8(16.00%) | 9(11.11%) |  |
| **Shape**, No. (%) |  |  |  | 0.817 |
| Irregular | 12(38.71%) | 18(36.00%) | 30(37.08%) |  |
| Round and oval | 19(61.29%) | 32(64.00%) | 51(62.96%) |  |
| **Lobulation**, No. (%) |  |  |  | 0.247 |
| Absent | 16(51.61%) | 18(36.00%) | 34(41.98%) |  |
| Presence | 15(48.39%) | 32(64.00%) | 47(58.02%) |  |
| **vacoule**, No. (%) |  |  |  | 0.795 |
| Absent | 24(77.42%) | 36(72.00%) | 60(74.07%) |  |
| Presence | 7(22.58%) | 14(28.00%) | 21(25.93%) |  |
| **Air-bronchogram**, No. (%) |  |  |  | 1.000 |
| Absent | 27(87.10%) | 43(86.00%) | 70(86.42%) |  |
| Presence | 4(12.90%) | 7(14.00%) | 11(13.58%) |  |
| **Pleural deformation**, No. (%) |  |  |  | 0.028 |
| Type a | 4(12.90%) | 14(28.00%) | 18(22.22%) |  |
| Type b | 7(22.58%) | 3(6.00%) | 10(12.35%) |  |
| Type c | 8(25.81%) | 8(16.00%) | 16(19.75%) |  |
| Type d | 12(38.71%) | 19(38.00%) | 31(38.27%) |  |
| Type e | 0(0.00%) | 6(12.00%) | 6(7.41%) |  |
| **CTv (HU)** | -575.57(-632.47, -518.47) | -647.94(-690.37, -605.85) | -618.26(-672.59, -577.62) | < 0.001 |
| **MD (mm)** | 16.80(13.20, 23.90) | 14.10(10.75, 18.43) | 14.80(11.50, 19.40) | 0.015 |
| **MVD (mm)** | 12.80(10.60, 17.30) | 10.45(7.98, 14.18) | 11.40(8.66, 15.65) | 0.015 |

P:The P value was calculated by comparing the IAC group and MIA group; Distance: The shortest distance between the largest invasive component area and the visceral pleura; Depth: Depth is defined as the depth of the largest invasive component area (the vertical distance between the point closest to the visceral pleura and the point farthest from the visceral pleura); CTv refers to the CT attenuation value on the maximum axial layer; MD refers to the maximum diameter on the maximum axial layer; MVD refers to the maximum vertical diameter of the maximum diameter on the maximum axial layer.

Table S3 Differential analysis between non-interlobar pleura and interlobar pleura according to the pathological components of the area adjacent pleura and CT manifestation.

| Variable | Non-interlobar pleura | | | |  | Interlobar pleura | | | |
| --- | --- | --- | --- | --- | --- | --- | --- | --- | --- |
|  | Alveoli/Lepidic (n= 32) | Acinar/Papillary (n= 16) | Total (n= 48) | P^δ^ value |  | Alveoli/Lepidic (n= 22) | Acinar/Papillary (n= 11) | Total (n= 33) | P^ε^ value |
| **Age**  Median (25th to 75th percentile) years | 58.50(48.25, 66.25) | 60.50(50.25, 62.75) | 59.50(49.00,64.00) | 0.930 |  | 58.00(45.00, 64.25) | 62.00(51.00, 66.00) | 60.00(50.05,64.50) | 0.534 |
| **Sex/Male**, No. (%) | 10(31.25%) | 3(18.75%) | 13(27.08%) | 0.497 |  | 7(31.82%) | 4(36.36%) | 11(33.33%) | 1.000 |
| **Smoking history**, No. (%) | 1(3.13%) | 0(0.00%) | 1(2.08%) | 1.000 |  | 3(13.64%) | 2(18.18%) | 5(15.15%) | 1.000 |
| **Pathological types**, No. (%) |  |  |  | < 0.001 |  |  |  |  | 0.005 |
| IAC | 7(21.88%) | 12(75.00%) | 19(39.58%) |  |  | 4(18.18%) | 8(72.73%) | 12(36.36%) |  |
| MIA | 25(78.13%) | 4(25.00%) | 29(60.42%) |  |  | 18(81.82%) | 3(27.27%) | 21(63.64%) |  |
| **Ki-67**, No. (%) |  |  |  | 0.307 |  |  |  |  | 0.559 |
| < 10% | 28(93.33%) | 11(78.57%) | 39(88.64%) |  |  | 20(95.24%) | 9(90.00%) | 29(93.55%) |  |
| ≥10% | 2(6.67%) | 3(21.43%) | 5(11.36%) |  |  | 1(4.76%) | 1(10.00%) | 2(6.45%) |  |
| Absence | 2 | 2 | 4 |  |  | 1 | 1 | 2 |  |
| **Distance**(mm), median (25th – 75th  percentile) | 1.75(1.00, 2.00) | 0.00(0.00, 0.00) | 1.00(0.00, 2.00) | < 0.001 |  | 1.25(0.500, 2.00) | 0.00(0.00,0.00) | 1.00(0.00, 2.00) | < 0.001 |
| **Depth**(mm) median (25th – 75th percentile) | 2.00(0.63, 3.75) | 6.00(4.25, 11.50) | 3.00(1.13, 6.00) | < 0.001 |  | 2.00(0.50, 3.25) | 7.00(4.00, 10.00) | 2.00(0.75, 7.00) | 0.004 |
| **Tumor location**, No. (%) |  |  |  | 0.563 |  |  |  |  | 0.154 |
| Right upper lobe | 11(34.38%) | 9(56.25%) | 20(41.67%) |  |  | 5(22.73%) | 6(54.55%) | 11(33.33%) |  |
| Right middle lobe | 2(6.25%) | 0(0.00%) | 2(4.17%) |  |  | 3(13.64%) | 2(18.18%) | 5(15.15%) |  |
| Right lower lobe | 5(15.63%) | 3(18.75%) | 8(16.66%) |  |  | 7(31.82%) | 2(18.18% | 9(27.27%) |  |
| Left upper lobe | 8(25.00%) | 3(18.75%) | 11(22.92%) |  |  | 6(27.27%) | 0(0.00%) | 6(18.18%) |  |
| Left lower lobe | 6(18.75%) | 1(6.25%) | 7(14.58%) |  |  | 1(4.54%) | 1(9.09%) | 2(6.06%) |  |
| **Shape**, No. (%) |  |  |  | 0.757 |  |  |  |  | 0.714 |
| Irregular | 12(37.50%) | 5(31.25%) | 17(35.42%) |  |  | 8(36.36%) | 5(45.45%) | 13(39.39%) |  |
| Round and oval | 20(62.50%) | 11(68.75%) | 31(64.58%) |  |  | 14(63.64%) | 6(54.55%) | 20(60.61%) |  |
| **Lobulation**, No. (%) |  |  |  | 0.357 |  |  |  |  | 0.163 |
| Absent | 11(34.38%) | 8(50.00%) | 19(39.58%) |  |  | 8(36.36%) | 7(63.64%) | 15(45.45%) |  |
| Presence | 21(65.63%) | 8(50.00%) | 29(60.42%) |  |  | 14(63.64%) | 4(36.36%) | 18(54.55%) |  |
| **vacoule**, No. (%) |  |  |  | 0.729 |  |  |  |  | 0.696 |
| Absent | 24(75.00%) | 13(81.25%) | 37(77.08%) |  |  | 16(72.73%) | 7(63.64%) | 23(69.70%) |  |
| Presence | 8(25.00%) | 3(18.75%) | 11(22.92%) |  |  | 6(27.27%) | 4(63.64%) | 10(30.30%) |  |
| **AirBronchogram**, No. (%) |  |  |  | 0.079 |  |  |  |  | 0.586 |
| Absent | 25(78.13%) | 16(100.00%) | 41(85.42%) |  |  | 20(90.91%) | 9(81.82%) | 29(87.88%) |  |
| Presence | 7(21.87%) | 0(0.00%) | 7(14.58%) |  |  | 2(9.09%) | 2(18.18%) | 4(12.12%) |  |
| **Pleural deformation**, No. (%) |  |  |  | 0.019 |  |  |  |  | 0.933 |
| Type a | 8(25.00%) | 6(37.50%) | 14(29.17%) |  |  | 3(13.64%) | 1(9.09%) | 4(12.12%) |  |
| Type b | 5(15.62%) | 1(6.25%) | 6(12.50%) |  |  | 2(9.09%) | 2(18.18%) | 4(12.12%) |  |
| Type c | 10(31.25%) | 2(12.50%) | 12(25.00%) |  |  | 3(13.64%) | 1(9.09%) | 4(12.12%) |  |
| Type d | 3(9.38%) | 7(43.75%) | 10(20.83%) |  |  | 14(63.63%) | 7(63.64%) | 21(63.64%) |  |
| Type e | 6(18.75%) | 0(0.00%) | 6(12.50%) |  |  | 0(0.00%) | 0(0.00%) | 0(0.00%) |  |
| **CTv (HU)** | -627.04(-673.46, -581.82) | -581.11(-682.67, -525.58) | -613.41(-673.46, -577.60) | 0.168 |  | -635.01(-682.67, -607.66) | -599.28(-634.82, -518.47) | -623.49(-670.03, -567.26) | 0.040 |
| **MD (mm)** | 14.10(11.33, 18.38) | 18.25(14.15, 24.68) | 15.20(11.95,20.30) | 0.030 |  | 13.30(10.65, 15.70) | 18.20(14.30,23.20) | 14.30(11.45,19.25) | 0.015 |
| **MVD (mm)** | 10.35(7.85,14.95) | 16.05(11.25, 19.38) | 11.85(8.00,16.18) | 0.024 |  | 10.20(8.80, 12.93) | 14.00(10.90, 16.10) | 11.20(9.15, 14.35) | 0.019 |

P^δ^: The P^δ^ value was calculated by comparing the Alveoli/Lepidic group and Acinar/Papillary group related to Non-interlobar pleura; P^ε^: The P^ε^ value was calculated by comparing the Alveoli/Lepidic group and Acinar/Papillary group related to Interlobar pleura; P^ζ^:The P^ζ^ value was calculated by comparing the IAC group and MIA group; Distance: The distance between invasive components area and Non-interlobar pleura; Depth: Depth is defined as the vertical distance between the point closest to the Non-interlobar pleura and the point farthest from the Non-interlobar pleura; CTv refers to the CT attenuation value on the maximum axial layer; MD refers to the maximum diameter on the maximum axial layer; MVD refers to the maximum vertical diameter of the maximum diameter on the maximum axial layer.
